# Developmental regulation of MURF E3 ubiquitin ligases in skeletal muscle

**DOI:** 10.1007/s10974-012-9288-7

**Published:** 2012-03-17

**Authors:** Sue Perera, Baljinder Mankoo, Mathias Gautel

**Affiliations:** 1 Cardiovascular Division, BHF Centre of Research Excellence, King’s College London, London, UK; 2 Randall Division for Cell and Molecular Biophysics and Cardiovascular Division, King’s College London, New Hunt’s House, Guy’s Campus, London, SE1 1UL UK

**Keywords:** MURF, p62, SQSTM1, NBR1, Myofibrils, Microtubules, Skeletal muscle development, Fibre-type differentiation, Slow fibres

## Abstract

The striated muscle-specific tripartite motif (TRIM) proteins TRIM63/MURF1, TRIM55/MURF2 and TRIM54/MURF3 can function as E3 ubiquitin ligases in ubiquitin-mediated muscle protein turnover. Despite the well-characterised role of MURF1 in skeletal muscle atrophy, the dynamics of MURF isogene expression in the development and early postnatal adaptation of skeletal muscle is unknown. Here, we show that MURF2 is the isogene most highly expressed in embryonic skeletal muscle at E15.5, with the 50 kDa A isoform predominantly expressed. MURF1 and MURF3 are upregulated only postnatally. Knockdown of MURF2 p50A by isoform-specific siRNA results in delayed myogenic differentiation and myotube formation in vitro, with perturbation of the stable, glutamylated microtubule population. This underscores that MURF2 plays an important role in the earliest stages of skeletal muscle differentiation and myofibrillogenesis. During further development, there is a shift towards the 60 kDa A isoform, which dominates postnatally. Analysis of the fibre-type expression shows that MURF2 A isoforms are predominantly slow-fibre associated, whilst MURF1 is largely excluded from these fibres, and MURF3 is ubiquitously distributed in both type I and II fibres.

**Electronic supplementary material:**

The online version of this article (doi:10.1007/s10974-012-9288-7) contains supplementary material, which is available to authorized users.

## Introduction

Protein turnover is a highly selective and tightly coordinated process. In skeletal muscle, where the rates of protein synthesis and degradation need to be carefully balanced under different physiological stimuli to achieve muscle plasticity, proteolysis is mediated mainly by the ubiquitin–proteasome system (UPS), and the autophagy/lysosomal system (reviewed in Sandri (2008)). Both pathways remove misfolded or damaged proteins, but also functional proteins, which must be simultaneously replaced by their related isoforms––such isoform switching is critical for the postnatal maturation and physiological adaptation of muscle (reviewed in Schiaffino et al. (2008)) but also for the maintenance of differentiated muscle (Masiero et al. 2009).

During UPS-mediated protein turnover, the targeted substrate is covalently modified by the attachment of the small peptide ubiquitin, via an enzymatic cascade whose specificity is determined by designated E3 ligases. Where, when and which protein is degraded therefore depends on the expression status of its dedicated E3 ligase. In muscle, multiple E3 ligases have been identified, known as atrogenes––of which the main ones are the F-box protein atrogin1/MAFbx and the MURF (muscle-specific ring finger)/TRIM (RING/B-box/coiled-coil or tripartite motif containing) family of proteins (reviewed in (Glass and Roubenoff 2010; Willis et al. 2009)).

The MURF family was first identified by the interaction of one of its members, MURF3, with serum response factor (SRF) in a yeast two-hybrid screen (Spencer et al. 2000). The three MURF genes known (MURF1/TRIM63; MURF2/TRIM55; MURF3/TRIM54) encode highly homologous proteins, which can homo- and hetero-dimerise via their coiled-coil domains (Centner et al. 2001). Differential splicing occurs in the C-termini of some MURF genes, producing isoforms with tissue-specific expression patterns, particularly evident in the case of MURF2, of which at least four isoforms are expressed in human and mouse (Perera et al. 2011; Pizon et al. 2002). At least three of these isoforms are expressed in skeletal muscle (MURF2 p60A, p60B and p50A), while a small splice variant, p27A, lacking the coiled-coil domain that mediates hetero- and homodimerisation of TRIM E3 ligases, is expressed in the heart of mice and men (Perera et al. 2011; Pizon et al. 2002). In contrast, MURF1 does not seem to be differentially spliced, and only one variant has been reported for MURF3 (Centner et al. 2001; Spencer et al. 2000). Interactions of MURF1 and MURF2 with domains near the C-terminus of titin lead to association with the M-band (Centner et al. 2001; Pizon et al. 2002). However, MURF1 and MURF2 and likely also MURF3 can translocate to the nucleus during atrophic stress in cardiomyocytes (Lange et al. 2005; Pizon et al. 2002) and in skeletal muscle in vivo (Lange et al. 2005; Ochala et al. 2011), where they appear to act as repressors of muscle gene expression. MURF1 and MURF3 have also been found at the Z-disk (Centner et al. 2001; Spencer et al. 2000), indicating that the sarcomeric targeting of MURFs is not only dependent on M-band titin interactions, and that atrophic signalling can strongly influence their localisation. In addition, MURF2 and MURF3 were shown to colocalise with stable glutamylated microtubules during myofibrillar assembly in vitro (Pizon et al. 2002; Spencer et al. 2000), and also at the earliest stages of cardiac myofibrillogenesis in vivo (Perera et al. 2011). During myogenic differentiation, stable arrays of glutamylated microtubules accumulate with a simultaneous reduction of the dynamic tyrosinated tubulin pool (Gundersen et al. 1989) and seem to be involved in the active transport of sarcomeric proteins to the sites of myofibrillogenesis (Pizon et al. 2005). Together, these observations suggest that the association of MURFs with microtubules could regulate their stability, facilitating muscle differentiation.

In the sarcomere, MURFs form an M-band associated signalling module together with titin kinase and its associated scaffolds nbr1 and p62/SQSTM1 (Lange et al. 2005), both of which participate in ubiquitin-related signalling, as well as in the control of autophagy (Bjorkoy et al. 2005; Kirkin et al. 2009; Komatsu et al. 2007; Waters et al. 2009). In autophagy, proteins targeted for degradation by ubiquitin modification usually bear lysine-63 linked polyubiquitin chains, and are recruited to autophagosomes containing lipidated LC3 at their membranes by specific adapter proteins binding both ubiquitin and LC3 (Geng and Klionsky 2008). Proteasomal degradation predominantly removes proteins labelled with lysine-48 linked chains. MURFs therefore not only associate with different cytoskeleton components such as microtubules, Z-disks and M-bands, as well as with metabolic enzymes and nuclear proteins, but seem to cooperate with diverse proteins implicated in selective protein degradation by the proteasome and autophagosome, and to target proteins of metabolic regulation, sarcomere assembly and transcriptional regulation (Lange et al. 2005; Witt et al. 2005).

Only for MURF1 has an association with mature type II (fast) fibres been shown (Moriscot et al. 2010), and combined ablation of MURF1 and MURF2 were reported to affect postnatal muscle growth, metabolism, and fibre-type differentiation (Moriscot et al. 2010; Witt et al. 2008). Whilst considerable data have emerged on the atrophy-related function of MURFs, with almost complete focus on MURF1, the expression profile of these highly cooperative and perhaps partly redundant proteins during skeletal muscle development and postnatal differentiation is surprisingly largely unknown. Therefore, we studied the expression profile of MURFs and their associated UPS/autophagy-linked binding partners during skeletal muscle development and probed the functions of MURF2 p50, the dominant MURF during development, by short-term siRNA-mediated knockdown.

## Materials and methods

### Analysis of MURF transcripts

RNA was extracted from freshly isolated tissues of healthy C57BL/6 mice using the RNeasy Mini Kit (Qiagen, UK). cDNA was prepared in a total reaction volume of 20 μl using 2 μg of total RNA, utilising the Super-Script III RT-PCR Kit (Invitrogen, UK). 2 μl of cDNA was amplified in a final 50 μl reaction. The primers used were:

- MURF1
- For 5′-CCGCTCGAGCCACCATGGATTATAAATCTAGCCTG,
- Rev 5′-TTTTTTGGATCCCCTTGGTGTTCTTCTTTACCCTC;
- MURF2
- For 5′-CCGCTCGAGCCACCATGAGCACTTCTCTGAATTACAAG,
- Rev 5′-TTTTCCCGGGCACCTTCATTTAGGGAATTCAACC;
- p62/SQSTM1
- For 5′-GGAGGAGCTCGAGCCATGGCGTTCACGGTGAA,
- Rev 5′-TATTATTTTTGGATCCTTCAATGGTGGAGGGTGTTCG;
- LC3B
- For 5′-CGGAGCTTTGAACAAAGAGTG,
- Rev 5′-GTCCCGAATGTCTCCTGCG;
- nbr1
- For 5′-ATGGAACCACAGGTTACTCTA,
- Rev 5′-GCAGAAGAACATTGCTCTGG.

For analysis of relative transcript levels by semi-quantitative RT-PCR, glyceraldehyde-3-phosphate (GAPDH) was used as a loading control, with the primers

- For 5′-GGCACTGTCAAGGCTGAAAACG
- Rev 5′- GGAGATGAGATGATACCACGCTTAG.

All reactions were done in triplicate and relative expression was quantified by Image-J software (National Institute of Health, USA).

### Western blotting

Snap-frozen tissue samples were homogenised by freeze-slamming, dissolved in SDS-sample buffer (3.7 M Urea, 134.6 mM Tris, 5.4 % SDS, 2.3 % NP-40, 4.45 % β-mercaptoethanol, 4 % glycerol, 6 mg/100 ml bromophenol blue, pH 6.8) and boiled for 2 min. Preparations were run on 10 or 12 % polyacrylamide minigels and separated proteins were either immediately visualised by Coomassie blue staining (loading) or blotted onto nitrocellulose Protran BA-83, (Schleicher & Schuell, Germany) overnight in transfer buffer (3 g Trizma base, 14.5 g glycine, 0.1 g SDS, 200 ml methanol) at 60 mA in a wet-blot transfer unit (Biorad, UK). Protein transfer was confirmed by Ponceau-S staining.

The membrane was blocked by incubating for 1 h in low-salt buffer (0.9 % NaCl, 0.1 % Tween-20, 50 mM Tris pH 7.4) supplemented with 5 % non-fat milk powder. The membrane was incubated in primary antibody for 2 h at room temperature or overnight at 4 °C; the blot was washed 3 × 5 min in low-salt buffer containing 1 % non-fat milk powder; and subsequently incubated in secondary antibody for 1 h at room temperature. Chemiluminescence reaction was performed using homemade developing solution (7.5 ml H_2_0, 1 ml 25 mM 5-amino-2,3-dihydro-1,4-phthalazinedione, 1 ml 5 mM 4-Iodophenol, 0.5 ml 1 M Tris pH 7.5) and visualised on Hyperfilm ECL (Amersham, USA). Densitometry was performed using ImageJ software (National Institute of Health, USA).

### Cell culture and immunofluorescence

For C2C12 transfections, approximately 1.5 μg of plasmid DNA was diluted in 30 μl of DMEM, 6 μl of Lipofectamine2000 (Invitrogen, UK) was separately mixed in another 30 μl of DMEM. The DNA and Lipofectamine2000 were mixed together and left to complex for 30–40 min. Cells were washed twice with DMEM. The DNA-Lipofectamine2000 mix was added first to the cells, swirled for 1 min, then 0.8 ml of DMEM was added per 35 mm culture dish. Cells were incubated for 4–6 h at 37 °C in 5 % CO_2_. Cells were changed to 1.5 ml of C2C12 differentiating medium. Cells were fixed with 4 %-paraformaldehyde (PFA)/PBS at required time points. If cells were intended for microtubule staining, they were washed twice with microtubule protection (MP) buffer (65 mM PIPES, 25 mM HEPES, 10 mM EGTA, 3 mM MgCl_2_, pH 6.9), and fixed with 4 % PFA/MP buffer. Samples were permeabilised with 0.2 % Triton X-100 in PBS for 5 min. Primary and secondary antibodies were diluted in Gold buffer (20 mM Tris-base, 155 mM NaCl, 2 mM EGTA, 2 mM MgCl_2_ pH 7.5) containing 5 % BSA. Cells were incubated in primary antibodies for 1 h at room temperature or overnight at 4 °C, then in secondary antibodies for 1 h at room temperature. Specimens were mounted in 0.1 M Tris–HCl pH 9.5-glycerol (3:7) including 50 mg/ml n-propyl-gallate as anti-fading reagent (Messerli et al. 1993) and viewed on a Zeiss LSM Meta 510 confocal microscope equipped with an Argon-laser, Helium–Neon-lasers and UV-diode.

### Cryosectioning and immunofluorescence

Perinatal and adult mouse gastrocnemius and soleus were freshly isolated, mounted in cryosectioning medium and immediately submerged in liquid isopentane in liquid nitrogen. Samples were then stored at −80 °C until cryosectioning. Before sectioning, samples were first placed in the cryostat cold chamber for 1 h to equilibrate. Samples were embedded and 10–20 μm sections were cut. Samples were allowed to dry for 1–2 h, then directly fixed with 4 % PFA/PBS for 2 min, permeabilised with 0.2 % Triton X-100 in PBS for 1 min, blocked for 30 min with normal horse serum (NHS) in 1 % BSA/gold buffer solution, and then incubated for 1 h at room temperature with primary antibody. After 3 × 5 min washes with PBS, secondary antibody was added for another 1 h at room temperature. Samples were mounted as previously described (Messerli et al. 1993).

### siRNA experiments

Isoform-specific 19-mer target siRNA were designed against mouse- and rat-specific sequences (identical for both species) that bridged the unique exon–exon boundaries for MURF2 p50A and p60A, which corresponded to exons 8–11 and exons 9–11 respectively, as obtained from the NCBI database. For p60A knockdown,

- FOR 5′-gatccccGCTACCTCTCAGATTGGATttcaagagaATCCAATCTGAGAGGTAGCtttttggaac-3′

and

- REV 5′-gttccaaaaaGCTACCTCTCAGATTGGATtctcttgaaATCCAATCTGAGAGGTAGCggggatc-3′ were used.

For p50A knockdown,

- FOR 5′-gatccccCTGGTGACACAGATTGGATttcaagagaATCCAATCTGTGTCACCAGtttttggaac-3′

and

- REV 5′-gttccaaaaaCTGGTGACACAGATTGGATtctcttgaaATCCAATCTGTGTCACCAGggggatc-3′ were used. For controls, scrambled sequences were used.

The H1-GFP vector used is based on pSuper ((Brummelkamp et al. 2002); H1 RNA-Pol III promoter) and pEGFP (Clontech; EGFP under the control of the CMV promoter). This ensures that the siRNA is expressed concurrently with GFP, which allows for easy identification of transfected cells. The forward and reverse primers for siRNA were dissolved in 50 μl of water; and self-annealed using 2 μl of each oligonucleotide and 46 μl of annealing buffer (100 mM potassium acetate, 30 mM HEPES–KOH pH 7.4, 2 mM magnesium acetate) to give double-stranded constructs. Construct specificity and knockdown efficiency was tested by transfecting the siRNA into COS-1 cells, along with a plasmid that expressed either GFP-mouse MURF2 p60A or p50A, and assessing the reduction of the specific target protein by Western blotting. For expression of these GFP-fusion proteins, full-length mouse MURF2 cDNA was PCR-amplified as explained previously, and the products were cloned into the pEGFP-C1 vector (Clontech). Western blots of knockdown efficiency were previously described (Perera et al. 2011). Statistical analysis was performed by GraphPad Instat3 software.

### Antibodies

For Western blotting, the commercial goat anti-MURF2A (Abcam ab4387, 1:500), rabbit anti-HPC (1:2,000, Pizon et al. 2002) and rabbit anti-HP60 (1:2,000, Pizon et al. 2002) antibodies were used (see Supplemental Fig. 1 for epitope localisations). Additionally, rabbit anti-p62/Sequestosome1 (Abcam ab56416, 1:500), rabbit anti-LC3B (Cell Signalling #2775, 1:500), mouse monoclonal anti-nbr1 (Abcam ab55474, 1:500), rabbit anti-SRF (Santa-Cruz SC13029, 1:500), goat anti-MuRF3 (Santa-Cruz SC50252, 1:500), rabbit anti-GAPDH (Abcam ab9485, 1:1,000) and rabbit anti-actin (Sigma A2066, 1:1,500) antibodies were used. As secondary antibodies, HRP-conjugated rabbit anti-goat IgG and goat anti-rabbit IgG from Calbiochem (1:1,000); and HRP-conjugated rabbit anti mouse IgG (Dako, 1:1,000) were used.

For immunofluorescence, MURF2 was visualised with the goat anti-MURF2A (1:100), rabbit anti-HPC (1:100), and rabbit anti-HP60 (1:200) antibodies. Additionally, rabbit anti-sarcomeric α-actinin, rat anti-all tubulin (Abcam, clone YOL1/34, 1:100), mouse anti-glutamylated α-tubulin (Synaptic systems, clone 1D5, 1:200), rat anti-tyrosinated tubulin (Abcam, clone YL1/2, 1:200), mouse anti-acetylated α-tubulin (Abcam ab24610, clone 6-11B-1, 1:100) and mouse anti-multi ubiquitin (Stressgen SPA-205E, clone FK2, 1:100) were used. The mouse anti-all MyHC A4.1025 and anti-slow MyHC A4.840 antibodies were kindly donated by Dr. S. Hughes (Hughes et al. 1993), whilst the mouse anti-myomesin B4 antibody was a gift of Dr. E. Ehler (Grove et al. 1984). For triple staining, combinations of Alexa488, Cy3 and Cy5-conjugated secondary antibodies were used. Cy3 anti-goat Ig, Cy3 anti-mouse Ig, Cy5 anti-mouse Ig, Cy5 anti-rabbit Ig, Cy3 anti-rabbit Ig, Cy5-anti rat Ig were purchased from Stratech Scientific, USA and the subclass specific FITC-anti mouse IgM (for A4.840) was purchased from Sigma, UK.

## Results

### MURF2 isoforms undergo pre- and postnatal switching and overall downregulation in developing skeletal muscle

**Fig. 1:**
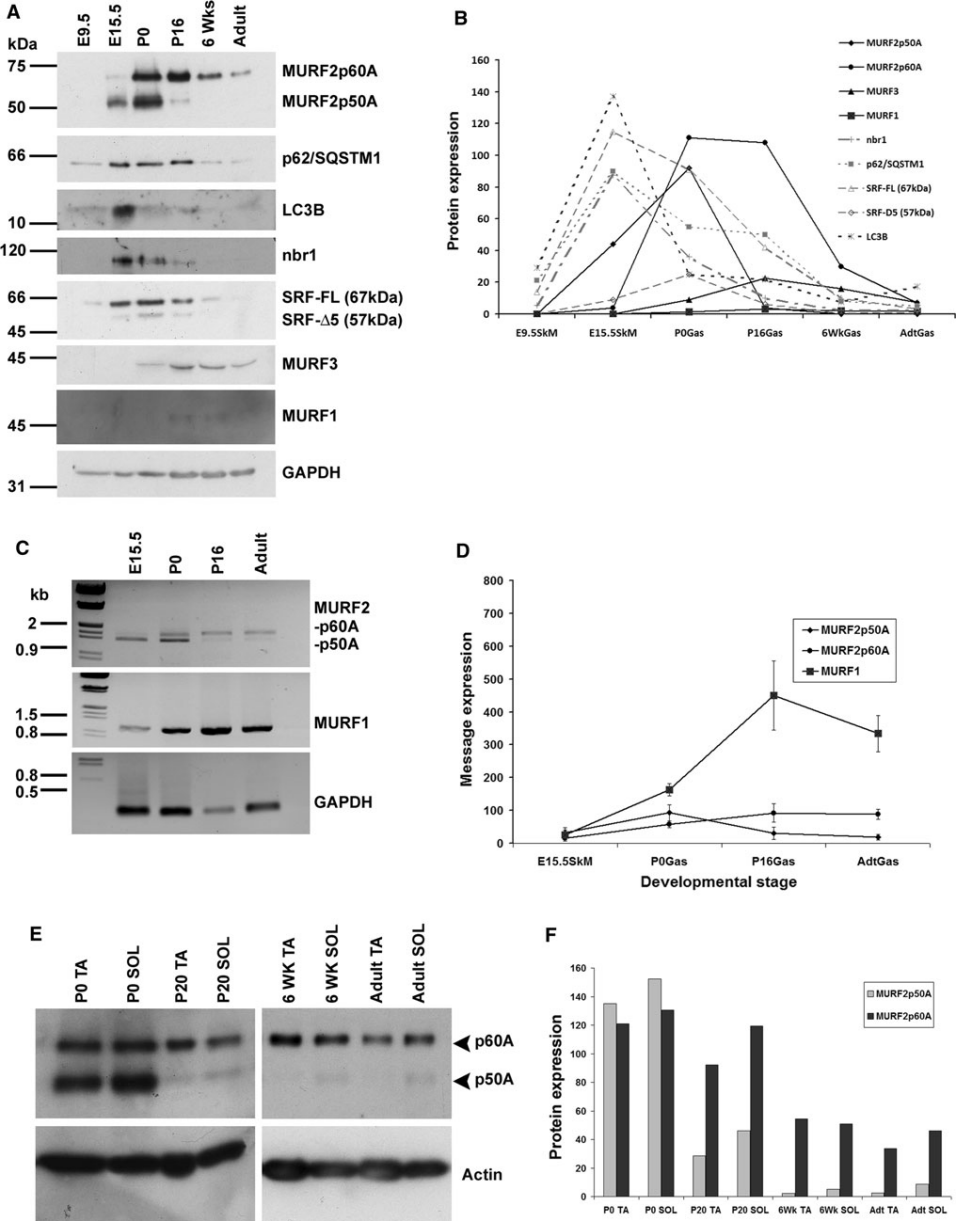
Developmental expression of MURF E3 ubiquitin ligases and related UPS/autophagy proteins in mouse skeletal muscle. **a** Western blot analysis of mouse skeletal muscle lysates (gastrocnemius) from embryonic (E) to postnatal (P) stages. **b** Plot of the densitometric analysis on Western blots normalised for GAPDH levels. **c** Semi-quantitative RT-PCR on cDNA isolated from mouse gastrocnemius. **d** Plot of semi-quantitative RT-PCR data obtained from three independent sets of experiments; *error bars* represent standard deviation. **e** Western blots showing the differential expression of MURF2 isoforms between predominantly fast (tibialis anterior––TA) and slow (soleus––SOL) muscle from P0 to adult stages. Note that p50 remains detectable postnatally only in soleus muscle

MURF2, which is alternatively spliced to generate multiple isoforms in both human and mouse (Perera et al. 2011; Pizon et al. 2002), is expressed in embryonic mouse skeletal mouse as early as E13.5–E15.5, as detected by the commercially available, goat anti-MURF2A-specific antibody, as two bands corresponding to the p60A and p50A isoforms (Supp. Fig. 1; Fig.1a). Identical results were obtained with a generic antibody with overlapping epitopes, HPC. During embryonic stages, the 50 kDa isoform dominates; however, there is a transition to the 60 kDa isoform during postnatal development, such that in adult gastrocnemius muscle, the p50A isoform can only be detected at very high exposures (Fig. 1a, Supplemental Fig. 2). The overall levels of both MURF2-A isoforms are reduced in adult skeletal muscle (Fig. 1a, b).

Given the highly homologous nature of MURF E3 ubiquitin ligases (Centner et al. 2001) and their predicted functions in muscle protein turnover (Willis et al. 2009), we explored the expression profile of all MURF family members and their functionally associated components of the UPS and autophagy/lysosomal systems. Protein levels of LC3B, nbr1 and p62/SQSTM1 peak at E15.5 and are then gradually downregulated (Fig. 1a, b). RT-PCR analysis shows a similar expression profile (Supplemental Fig. 3A-B), suggesting that ubiquitin- and autophagy-mediated protein turnover are tightly and co-ordinately regulated during early skeletal muscle development. SRF, a known target of MURF2 (Lange et al. 2005) and ligand of MURF3 (Spencer et al. 2000), and a mediator of muscle remodelling in response to hypertrophic stimuli (Carson and Booth 1998; Fluck et al. 2000; Gordon et al. 2001), is also expressed embryonically, but is surprisingly rapidly downregulated, possibly in parallel with increasing levels of MURF2 (Fig. 1a, b). MURF3 is only detected postnatally with the available antibody, implying that time-dependent regulation of the family of MURF E3 ubiquitin ligases could be functionally relevant (Fig. 1a, b). We also compared MURF1 and MURF2 message levels over time and found that although MURF1 is present embryonically, it is dramatically upregulated at birth and maintained at elevated levels thereafter (Fig. 1c, d). Taken together, these results suggest that MURF1 and MURF2 are dynamically regulated during embryonic skeletal muscle development, with MURF3 expression becoming notable only postnatally. The postnatal isoform switch in MURF2 and overall developmental downregulation mirrors previous observations during cardiac muscle differentiation (Perera et al. 2011).

### MURFs show fibre-type specific expression

As the establishment of fast and slow fibre subtypes is a key process during skeletal muscle development governing later muscle function, we asked whether MURFs exhibit fibre-type-specific expression.

Lysates of fast and slow muscles, tibialis anterior (TA) and soleus (SOL), were studied by Western blots, and densitometric analysis of these was performed. At P0, MURF2 p50A and p60A are abundant in both muscles; however as development proceeds, p50A levels are strongly downregulated in the TA, more so than in the soleus (Fig. 1e, f). In the adult, only trace amounts of p50A are detected in the TA, which is in contrast to the soleus that has approximately 3-fold higher expression levels of p50A (Fig. 1e, f). The adult soleus also shows noticeably higher levels of p60A compared to the TA (Fig. 1e, f). Given that slow fibres are absent in the adult TA, this suggests that MURF2 p50A is strongly slow-fibre-associated.

**Fig. 2:**
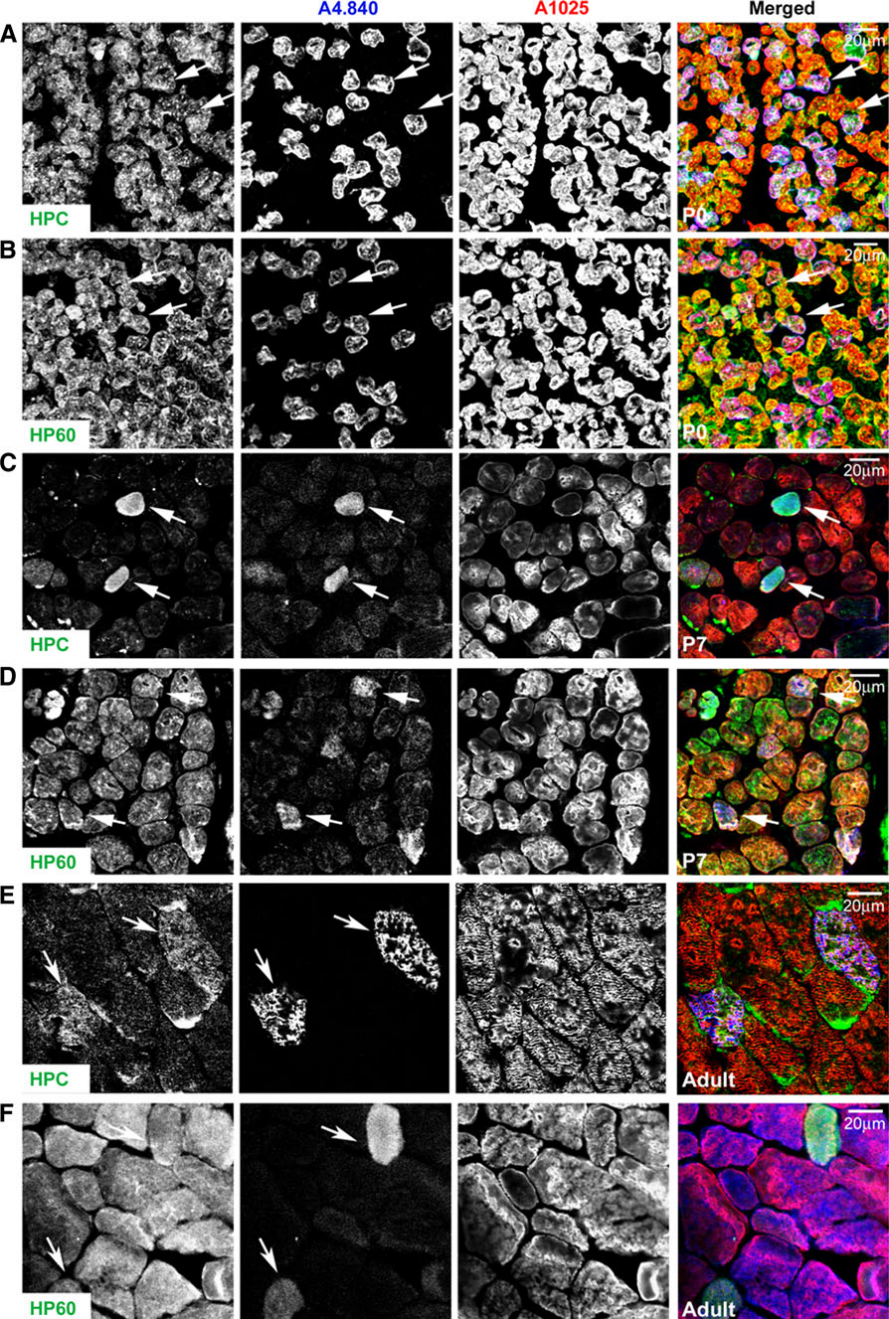
Fibre-type restriction of MURF2 expression. Cross-sections of gastrocnemius muscle were analysed by immunofluorescence microscopy. **a**, **b** MURF2 is ubiquitously present in all muscle fibres at P0, as detected by both the A-isoform specific HPC antibody and the p60A/B-specific HP60 antibody. **c** By P7, MURF2-A isoforms show slow-fibre expression. **d** At P7, the HP60 antibody shows that MURF2 p60A and B are more widely distributed. **e**, **f** In adult muscle, MURF2-A isoforms are again predominantly slow-fibre associated whilst HP60 staining gives a more widespread signal. Slow fibres were stained with monoclonal anti-slow myosin antibody A4.840, all myosin isoforms were visualised by the monoclonal anti- myosin antibody A1025. *Scale bars* 20 μm

To investigate this fibre-type specificity on the morphological level, immunofluorescence was performed on cryosectioned skeletal muscle (mixed fibre type, gastrocnemius). At P0, MURF2-A is ubiquitously present in all fibres (Fig. 2a, b) although slow fibres can already be detected by myosin staining in deeper regions of the muscle in agreement with previous results (Allen and Leinwand 2001). By P7 however, MURF2, as detected by the A-isoform specific HPC antibody, appears to be predominantly within slow fibres (Fig. 2c). Interestingly, the MURF2-p60A/B-specific HP60 antibody staining gives a more widespread signal at P7, which is possibly due to the MURF2 p60B isoform being ubiquitously distributed (Fig. 2d). In the adult, HPC-detected MURF2-A isoforms are again predominantly localised in slow fibres whilst the HP60 signal is again more homogeneous (Fig. 2e, f). This is in agreement with our Western blot data which shows that p50A is slow-fibre restricted and that p60A levels are also appreciably higher in slow muscle (Fig. 1f). It seems therefore that p60A levels are also more concentrated in slow fibres. In summary, MURF2-A isoforms are tightly developmentally regulated and p50A in particular assumes slow-fibre specific localisation during fibre subtype maturation, arguing for roles in both skeletal muscle differentiation and postnatal maturation.

**Fig. 3:**
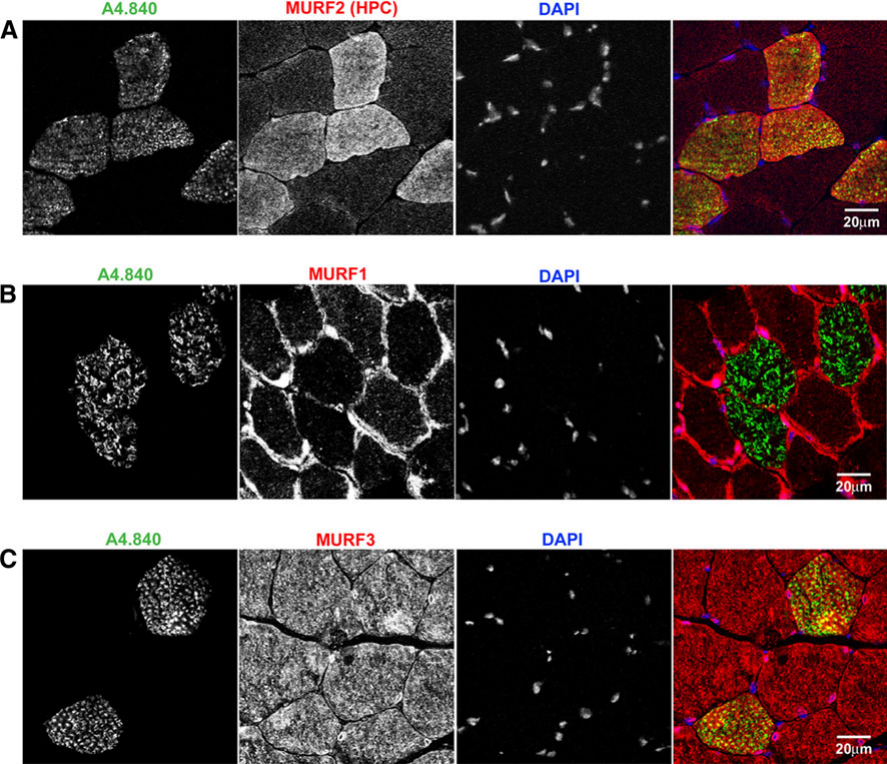
MURF family members have differential fibre-type-specific expression. **a** MURF2-A isoforms are strongly detected in type I/slow fibres stained by the monoclonal anti-slow myosin antibody A4.840. **b** MURF1 appears to be excluded from the slow fibres. **c** MURF3 is ubiquitously present in both fast and slow fibres. Cryosectioned samples of adult soleus were stained, and imaged by confocal microscopy. *Scale bars* 20 μm

MURF1 was recently shown to be fast (type II) fibre-associated in skeletal muscle, and a double knockout model (dKO) of both MURF1/2 appeared to shift the fibre balance towards slow (type I) fibres in the soleus (Moriscot et al. 2010). However, these authors did not investigate MURF2 or MURF3 expression; hence, we set out to fill this missing gap in the understanding of fibre-type specific MURF functions by comparing all three MURFs at the fibre level. Interestingly, whilst MURF2-A isoforms are highly expressed in the slow myosin-positive fibres in adult soleus (Fig. 3a), MURF1 is largely excluded from these slow fibres (Fig. 3b) in agreement with (Moriscot et al. 2010), while MURF3 is ubiquitously detected in all fibre types (Fig. 3c).

### The isoform regulation of MURF2 is conserved in vitro and in vivo during skeletal muscle differentiation

**Fig. 4:**
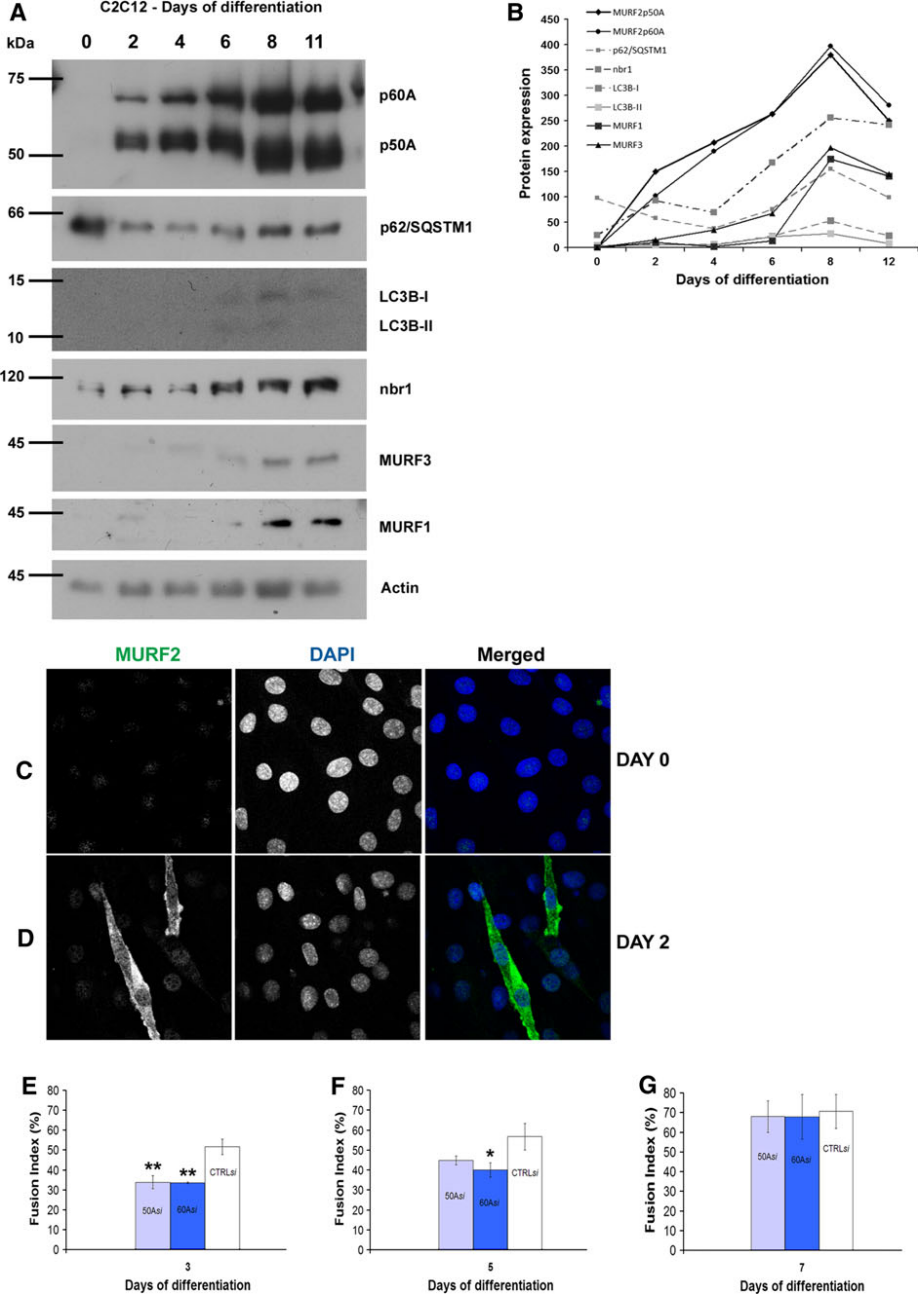
MURF expression is consistent both in vivo and in vitro and loss of MURF2 delays myogenic differentiation. **a** Analysis of MURFs and related UPS- and autophagy proteins in the murine C2C12 cell line as differentiation progressed. **b** Densitometric analysis of the Western blots normalised to GAPDH levels. **c** MURF2 is not detected in dividing myoblasts in vitro. **d** Serum withdrawal results in dramatic upregulation of MURF2-A isoforms at day 2 of myoblast differentiation. **e** SiRNA knockdown of MURF2 p50A or p60A results in significantly reduced fusion indexes for differentiating myoblasts. Myoblasts were considered to be differentiated if they had multiple nuclei and made cell-to-cell contacts with neighbouring cells. By day 3, degree of differentiation in cultures transfected with p50A- or p60A-specific siRNA were significantly lower than controls (*p* < 0.01). **f** By day 5, these cells were recovering and catching up with controls, however p60A-knockdown cultures still lagged behind controls (*p* < 0.05). **g** By day 7, control and siRNA-transfected myotubes possess similar degrees of differentiation. Data given as the mean percentage of transfected fused myotubes, obtained by counting >40 myoblasts from three sets of experiments. Error bars represent standard deviation. Statistical analysis was done by One-way ANOVA

Whilst these data provided a comprehensive understanding of the in vivo developmental regulation of MURFs, it was nevertheless important to verify that the expression profile was consistent in in vitro models for experimental purposes. We studied the expression pattern of MURFs and their related UPS- and autophagy-linked proteins in the mouse-derived C2C12 cell line as differentiation progressed, and found that MURFs are not expressed in proliferating myoblasts, however upon serum withdrawal, all three MURF isogenes are induced (Fig. 4a, b). It is noteworthy, however, that both message and protein levels of MURF1 and MURF3 lag behind those of MURF2 (Fig. 4a, b). All three MURFs are consistently upregulated during differentiation until day 8, after which their levels decline (Fig. 4a, b). The sequential appearance of MURF2 p50A before p60A seen in vivo (Fig. 1) is less pronounced in cultured cells, however; both isoforms are more or less co-expressed after day 2. The cardiac MURF2 p27A isoform was not detectable. As by immunofluorescence analysis, MURF2-A isoforms are again absent at day 0 (Fig. 4c) but are strongly induced in the cytoplasm of differentiating cells by day 2 (Fig. 4d). In agreement with all previous results, MURF2 could be detected in differentiating myoblasts in the cytoplasm, but the nuclei showed no significant staining.

### Knockdown of MURF2 isoforms leads to delayed myogenic differentiation in vitro

We therefore asked whether MURF2 isoforms had distinct roles during skeletal muscle differentiation, by employing a small interfering RNA approach (Brummelkamp et al. 2002), specifically targeting either the p50A or p60A isoforms (described in (Perera et al. 2011)). The siRNAs were expressed from a modified H1-GFP plasmid (Brummelkamp et al. 2002) that also encodes for GFP to enable identification of transfected cells. Due to the highly limited target sites for isoform-specific knockdown at the splice boundaries, only one siRNA could be designed for MURF2 p50 or p60 knockdown. However, our previous results showed that the cellular effects of these siRNA in neonatal cardiomyocytes could be rescued by transfection with siRNA-resistant MURF2 p50 cDNA (Perera et al. 2011). Proliferating myoblasts were transfected with the siRNAs or a scrambled control siRNA, then serum was withdrawn and the cells were allowed to differentiate. After 3 days, cultures transfected with either p50A- or p60A-specific siRNA showed significantly reduced levels of myoblast fusion and myotube formation (Fig. 4e, *p* < 0.001). After 5 days, these myoblasts appeared to recover, as the extent of fusion and myotube differentiation is noticeably increased, although still lagging behind control cultures (Fig. 4f, *p* ≤ 0.005). Analysis after 1 week demonstrated that the differentiation of cultures transfected with either isoform-specific or control siRNA are comparable (Fig. 4g), possibly because of gradual loss of the siRNA plasmid, or upregulation of compensatory MURF isogenes. The observation that both MURF2 p60 and p50 siRNAs had comparable effects, compared to control siRNA, agrees with the roughly equal expression levels of both isoforms (Fig. 4a).

### Knockdown of MURF2 disrupts de novo myofibrillogenesis in differentiating myoblasts

**Fig. 5:**
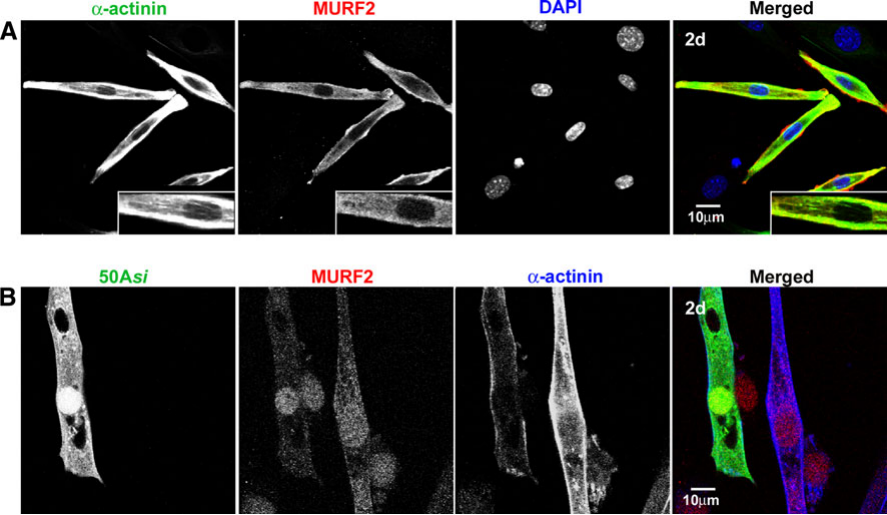
siRNA knockdown of MURF2 p50A leads to sarcomeric disassembly in vitro. **a** MURF2 and α-actinin (Z-disk marker) are strongly upregulated in differentiating myoblasts at day 2 of serum withdrawal. The *2-fold magnified insert* shows α-actinin appearing in the first cytoskeletal structures. Note that neighbouring, non-differentiating cells do not express MURF2 and α-actinin, only their nuclei are visible. **b** Knockdown of MURF2 p50A in differentiating C2C12 cells results in reduced α-actinin levels and organisation at 2 days. *Scale bars* 10 μm

We next considered whether the lag in differentiation observed in the C2C12 cell line would be reflected at the cellular level by staining for sarcomeric α-actinin, a Z-disk marker, which is one of the first sarcomeric proteins to be expressed in differentiating myoblasts and also one of the first components to be integrated into organised sarcomeric complexes during myofibrillogenesis (Sanger et al. 2005, 2010). As expected, in control cells at day 2 of differentiation, both α-actinin and MURF2 expression is strongly upregulated in myoblasts that have started to differentiate, elongate and fuse (Fig. 5a). However, in myoblasts transfected with p50A-specific siRNA, α-actinin staining is reduced both after 2 (Fig. 5b) and 5 days of differentiation (not shown). These observations suggest that cytoskeletal remodelling during myofibril assembly is perturbed by the short-term knockdown of MURF2 p50A. We therefore asked whether this affected also the transient scaffolds of myofibril assembly, the microtubule network.

### Loss of MURF2 is associated with perturbed stable microtubules in differentiating myoblasts

**Fig. 6:**
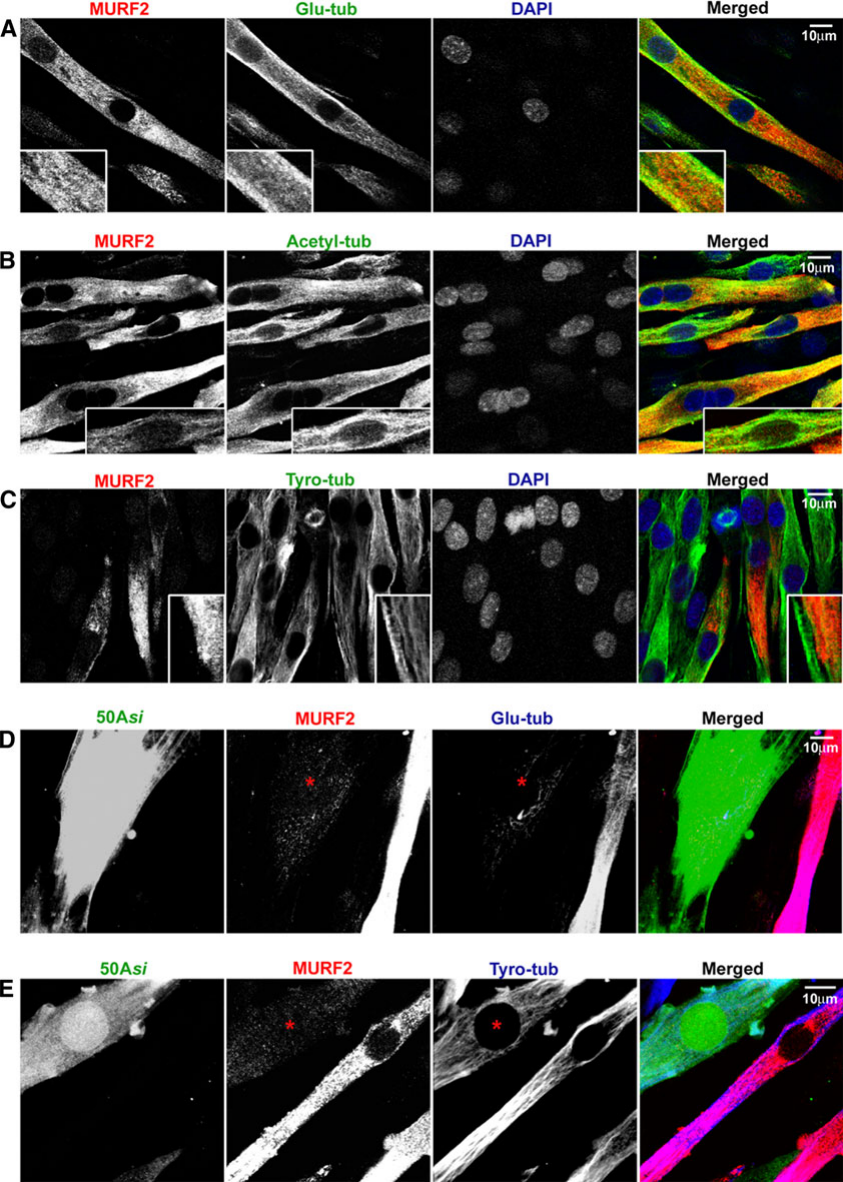
MURF2 knockdown disrupts microtubule post-translational modifications in differentiating myoblasts in vitro. **a**, **c** MURF2 is strongly colocalised with the glutamylated and acetylated microtubule populations (stable) in differentiating C2C12 cells, but not with the dynamic tyrosinated fraction. *Inserts* in **a**, **b**: ×2 magnified regions showing colocalisation (*yellow in overlay*) with glutamylated and acetylated microtubules but not the tyrosinated fraction. **d** Knockdown of p50A leads to decreased levels and organisation of the stable, glutamylated microtubule population in myoblasts at day 2 of differentiation. The image is overexposed to show the weak residual staining for glutamylated tubulin. **e** In contrast, the dynamic, tyrosinated microtubule pool appears unaffected by loss of p50A. *Scale bars* 10 μm

Myogenic differentiation is characterised by the formation of stable arrays of glutamylated tubulin, with a simultaneous reduction in the dynamic pool of tyrosinated tubulin (Gundersen et al. 1989); and MURF2 was previously shown to associate with microtubules in a skeletal muscle cell line prior to the formation of mature myofibrils (Pizon et al. 2002). In addition, it was recently shown that MURF2 colocalises with glutamylated microtubules also in vivo at the earliest stages of cardiac differentiation. Lastly, knockdown of MURF2 in neonatal rat cardiomyocytes perturbed their stable microtubule population (Perera et al. 2011). This led us to hypothesise that the observed delay in myotube formation and de novo myofibrillogenesis may at least in part be due to disrupted microtubule organisation. Hence, we looked at both the stable and dynamic fractions of microtubules during myoblast differentiation in vitro. We found that MURF2 is strongly colocalised with stable glutamylated and acetylated microtubules (Fig. 6a, b), but not with the dynamic tyrosinated population (Fig. 6c), which agrees well with previous findings in cardiomyocytes. We then looked at myoblasts transfected with p50A-specific siRNA. Here, the stable, glutamylated microtubules are noticeably reduced and are also not organised in parallel arrays (Fig. 6d), whereas the dynamic tyrosinated microtubule pool seemed unaffected (Fig. 6e). Taken together, our data support the conclusion that MURF2 functions in the earliest steps of myoblast differentiation and myofibrillar assembly, possibly via the ubiquitylation of microtubule-modifying complexes, which are crucial for achieving and/or maintaining the required stability of microtubules.

## Discussion

While the widely established function of the UPS and autophagy/lysosomal systems is proteolytic breakdown associated with catabolic muscle mass loss (Glass 2005), it is now becoming apparent that this is an over-simplification of a highly complex, tightly regulated process, which is also important during early development, for the ordered protein turnover required for muscle plasticity (Sandri 2010; Schiaffino et al. 2008), or during stress response (Willis et al. 2009, 2010).

The recent identification of muscle E3 ubiquitin ligases that target key cardiac transcription factors such as Nkx2.5 or phosphorylated c-Jun, which are critical to cardiac development and homeostasis, highlights the importance of the UPS in the earliest stages of striated muscle differentiation (Jang et al. 2011) and in stress response (Li et al. 2011). Furthermore, the discovery that the widely expressed E3 ubiquitin ligase RNF13 (whose levels are strongly reduced during skeletal muscle development) is important in limiting myoblast proliferation also underscores the relevance of this system to the regulation of skeletal muscle differentiation (Zhang et al. 2010). Dedicated E3 ligases therefore play roles far more complex than just in the induction of an atrophy programme of muscle protein degradation. Indeed, combined knockout mice for MURF1/2 (Witt et al. 2008) or MURF1/3 (Fielitz et al. 2007) show both pre- and postnatal phenotypes that indicate crucial and only partly redundant roles of MURF- mediated ubiquitylation of metabolic, sarcomeric and signalling proteins during muscle growth and homeostasis.

Similarly, an essential role of autophagy in early embryo differentiation has been identified using Atg5−/− mice (Tsukamoto et al. 2008). Later stages of mammalian embryonic development also require autophagy for controlling cell numbers, as mice with a targeted deletion of the autophagy-related gene beclin1 die between E7.5 and 8.5 (Yue et al. 2003). Postnatal inhibition of autophagy in skeletal muscle also promotes accumulation of toxic proteins, and surprisingly does not protect from atrophy, but instead causes muscle loss (Masiero et al. 2009; Masiero and Sandri 2010). These findings suggest that although yet poorly understood, the UPS and autophagy/lysosomal systems of proteolytic turnover are also essential regulators of development, maintenance and remodelling in muscle.

Whilst the atrophy-related function of MURFs has been well documented (reviewed in (Foletta et al. 2011; Sandri 2008), their roles in development are largely ignored. We have previously shown that MURF2 is the earliest MURF isogene to be expressed in differentiating myocardium, and that its targeted knockdown in cardiomyocytes results in disruption of sarcomere formation (Perera et al. 2011). Loss of MURF2 can be partly compensated by upregulation of MURF3, but compound loss of MURF2/MURF3 by siRNA knockdown in cultured cardiomyocytes produced a more severe myofibrillar phenotype than the individual knockdown of either isogene, suggesting partial compensation afforded in vitro by these homologues (Perera et al. 2011). MURF2 was found to be closely associated with the stable, glutamylated microtubule population important for myogenic differentiation (McElhinny et al. 2004; Perera et al. 2011; Pizon et al. 2002), a property observed also for MURF3 (Spencer et al. 2000), which might explain why MURFs might be required also during early skeletal muscle formation. We therefore analysed the expression profile of MURFs and the functionally associated autophagy/lysosomal machinery during skeletal muscle development. Our results show that these UPS and autophagy components are expressed at the earliest detectable stages of differentiated skeletal muscle, with MURF2 being the predominant MURF in the embryonic limb until birth. Unlike in the heart (Perera et al. 2011), MURF expression is not detected in the limb at E9.5, as myogenic differentiation of limb muscle lags behind the heart. From birth onwards, MURF1 and MURF3 levels are increased, whilst MURF2 gets downregulated, suggesting that the different MURF genes have time-dependent functions in skeletal muscle. Our observation that SRF, a known target of MURF2 (Lange et al. 2005) and an important regulator of postnatal growth (reviewed in (Braun and Gautel 2011; Charvet et al. 2006; Miano 2010), is reduced concomitantly with increasing MURF2 levels, suggests tentatively that MURFs might participate in protein homeostasis also on the transcriptional level during developmental and postnatal muscle remodelling. Fibre populations expressing slow but not neonatal MyHC are detectable prenatally in the rat. These differ from fibres expressing neonatal but not slow MyHC, which ultimately become fast fibres (Condon et al. 1990). However, the slow-fibre association of MURF2 appears after the determination of the contractile phenotype [as shown by slow myosin staining; (Allen and Leinwand 2001; Fladby and Jansen 1990)], suggesting that this switch is transcriptionally controlled upstream of MURF2.

Interestingly, we observed that MURFs display variable fibre-type preferences: while MURF2 is predominantly slow-fibre associated, MURF1 is largely excluded from these fibres and MURF3 is ubiquitously present. Furthermore, MURF2 was downregulated postnatally, quite dramatically in the case of p50A, and the switch from the 50 kDa isoform to the 60 kDa isoform was complete by postnatal day 16, which coincides with fibre-type maturation in skeletal muscle (Schiaffino 2010; Wirtz et al. 1983). MURF1 was recently shown to be predominantly fast (type II) fibre-associated in skeletal muscle (Moriscot et al. 2010), and we show here that MURF2A is, in contrast, preferentially expressed in slow, type I fibres. These results suggest that MURF isogenes, although highly homologous, by virtue of their variable expression pattern in different fibre types have access to specific molecular targets, which may be relevant to the maintenance of different fibre identities. This might be supported by the observation that MURF1/2 double knockout animals show a marked loss of type-II fibres in soleus muscle (Moriscot et al. 2010). However, the apparent presence of MURF2 p60B (Fig. 2d, f), and MURF3 (Fig. 3c) in all fibre types complicates the observed phenotypes, and further analysis would ideally require a specific antibody (currently unavailable) against the highly hydrophobic C-terminus of the p60B isoform.

Our observations prompt some revision of current assumptions about MURF function. MURF1 is widely regarded as a marker of muscle atrophy, due to its early and robust upregulation when muscle catabolism is triggered by disuse, denervation, starvation, sepsis or steroid administration (Glass and Roubenoff 2010; Sandri 2008). Indeed, MURF1 was confirmed as a major factor in muscle atrophy when knockout mice for MURF-1 were shown to be protected from denervation-induced atrophy (Bodine et al. 2001). It is currently not known whether MURF2 or MURF3 knockout mice are protected from atrophy, possibly in a manner restricted to slow fibres in the case of MURF2. However, as atrophic fibres revert to a more glycolytic, fast-fibre phenotype (Schiaffino et al. 2007), the atrophy-related upregulation of MURF1 might therefore reflect as much the switch in fibre type as the atrophic process itself. Furthermore, the assumption that MURF1 and 2 target redundant sets of muscle proteins (Witt et al. 2008) seems to be contradicted by the different phenotypes of the available dKO models, and requires complementing future insight into their fibre-type specific substrates or regulation mechanisms.

The interaction of MURF1/2 with myozenin-1 was proposed as a mechanism by which type-II fibres could be lost, as myozenins acts as a brake on calcineurin activity, and MYOZ2 knockout mice show an excess of slow skeletal muscle fibres (reviewed in (Gautel 2008)). This interpretation is also compatible with the observation that the combined knockout of MURF1/2 also leads to postnatal hypertrophic muscle growth (Witt et al. 2008).

Taken together, these results suggest that MURFs may participate in establishing and/or maintaining fibre-type identities. The exact mechanism responsible is unclear, but given that MURFs interact with multiple mitochondrial and glycolytic enzymes, sarcomeric proteins, scaffold proteins and transcription factors (Lange et al. 2005; Witt et al. 2005), altered energy metabolism and transcriptional or post-translational regulation of contractile properties are plausible pathways.

MURF2 was previously proposed to function as a transient “adaptor” in myofibrillogenesis in a skeletal muscle cell model (Pizon et al. 2002). The recently confirmed association of MURF2 with stable microtubules during cardiac myofibrillogenesis in vivo (Perera et al. 2011) further confirmed that MURFs play an active role in microtubule-mediated sarcomere assembly. Our knockdown experiments with two different MURF2 siRNA in C2C12 myoblasts show delayed myotube formation in MURF2-lacking cells with associated disruption of stable glutamylated microtubules, but the dynamic fraction of tyrosinated microtubules remained unaffected. This is intriguing, because MURF3 has also been shown to associate with glutamylated microtubules during skeletal muscle development (Spencer et al. 2000). However, the upregulation of MURF3 lags behind that of MURF2 (Fig. 1), and full functional compensation in early myoblasts might therefore not be possible. It is also conceivable that these isogenes therefore cooperate in their microtubule-binding roles, possibly via hetero-dimerisation. This hypothesis is further supported by previous observations that MURF2 and MURF3 show partial functional compensation in cardiomyocytes in vitro (Perera et al. 2011). Surprisingly, studies to date have not reported any obvious primary myofibrillar defects in single MURF2−/− mice (Willis et al. 2007); this might again be due to functional compensation by other MURF isogenes. The identities of the microtubule-associated enzymes performing microtubule modifications are only just beginning to emerge; we speculate that controlled ubiquitylation of such complexes might regulate microtubule stability and therefore, allows myogenic differentiation and sarcomerogenesis to proceed correctly.

In summary, we show that MURF ubiquitin E3 ligases are tightly developmentally regulated in skeletal muscle, with the p50A isoform of MURF2 being predominant at embryonic stages; MURF1, MURF2 p60A, and MURF3 play roles mostly in postnatal stages. MURF1 and MURF2 show fibre-type preference (MURF1 fast, MURF2A slow), which argues for functional specialisation and a role in defining the contractile and metabolic properties of these muscles. Ablation of MURF2 disrupts early stages of sarcomere assembly in the C2C12 skeletal muscle model, probably via perturbation of microtubule modifications important for the establishment of a stable microtubule network. Whilst it is important to realize that short-term effects in isolated cells in culture may not necessarily be reflected at the whole organism level, where multiple compensatory mechanisms act over longer durations, the study of dual MURF2/MURF3 knockout animals could be informative in deciphering the degree of cooperation and redundancy built into this highly specialised and multifunctional protein family.

## Electronic supplementary material

Below is the link to the electronic supplementary material.

Supplementary material 1 (PDF 956 kb)
