# Supplementary material for: Developmental regulation of MURF E3 ubiquitin ligases in skeletal muscle
Source: J Muscle Res Cell Motil. 2012 Mar 17;33(2):107–22. doi: 10.1007/s10974-012-9288-7 (PMC3353113; doi:10.1007/s10974-012-9288-7)

Supplementary information

Developmental regulation of MURF E3 ubiquitin ligases in skeletal muscle

Sue Perera[1,2], Baljinder Mankoo[2] and Mathias Gautel[1,2*]

[1] King's College London BHF Centre of Research Excellence,
[2]Randall Division for Cell and Molecular Biophysics and Cardiovascular Division, New Hunt's House, Guy's Campus, London SE1 1UL, United Kingdom

Supplementary Figure 1: Isoform splicing pattern, domain organisation and epitope localisation of MURF2.

The known splice variants of MURF2 with their domains are shown, and arrows indicate the positions of isoform-specific antibody epitopes used in this study. HPC recognises the p27A (only in heart), p50A and p60A isoforms, while HP60 recognises only the two 60 kDa splice variants.

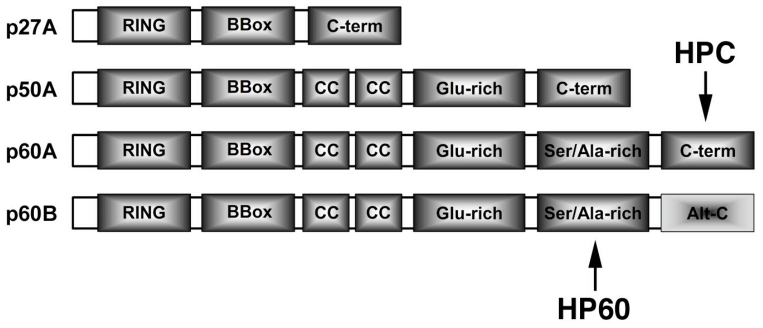

Supplementary Figure 2: Developmental downregulation of MURF2 in skeletal muscle.

Western blot analysis of mouse skeletal muscle (gastrocnemius) from embryonic to adult stages, showing the prenatal and postnatal isoform switch from p50 to p60, and overall postnatal down-regulation of MURF2. Note that p50 is not significantly expressed in adult gastrocnemius, which is dominated by fast fibres.

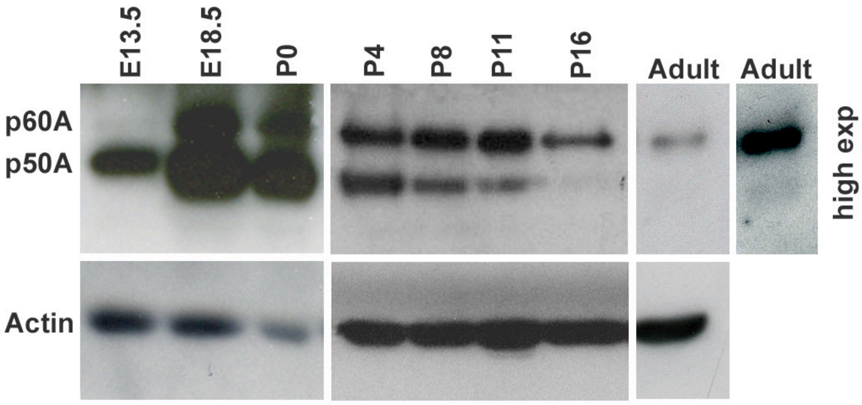

Supplementary Figure 3: RT-PCR analysis of autophagy-linked proteins.

A. RT-PCR analysis of nbr1, p62/SQSTM1 and LC3B transcripts during skeletal muscle development in the mouse using gastrocnemius samples. **B**. Densitometry of the message levels normalised to GAPDH.

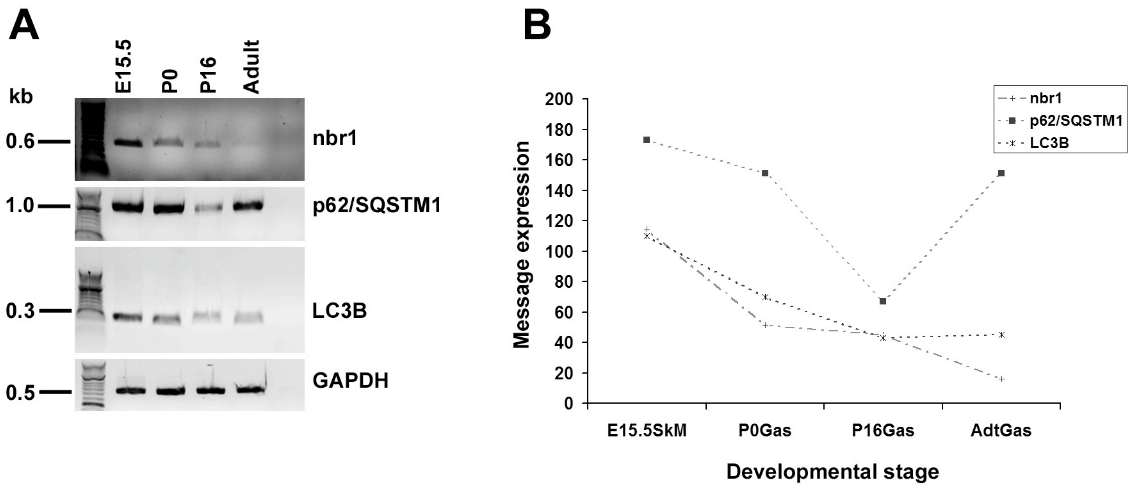

Supplement: Supplementary file 1 — Supplementary material 1 (PDF 956 kb) [file 10974_2012_9288_MOESM1_ESM.pdf]
